# A PET/CT approach to spinal cord metabolism in amyotrophic lateral sclerosis

**DOI:** 10.1007/s00259-016-3440-3

**Published:** 2016-07-15

**Authors:** Cecilia Marini, Angelina Cistaro, Cristina Campi, Andrea Calvo, Claudia Caponnetto, Flavio Mariano Nobili, Piercarlo Fania, Mauro C. Beltrametti, Cristina Moglia, Giovanni Novi, Ambra Buschiazzo, Annalisa Perasso, Antonio Canosa, Carlo Scialò, Elena Pomposelli, Anna Maria Massone, Maria Caludia Bagnara, Stefania Cammarosano, Paolo Bruzzi, Silvia Morbelli, Gianmario Sambuceti, Gianluigi Mancardi, Michele Piana, Adriano Chiò

**Affiliations:** 1 CNR Institute of Bioimages and Molecular Physiology, Milan, Section of Genoa Italy; 2 Nuclear Medicine, IRCCS San Martino IST, and Depth of Health Science, University of Genoa, Genoa, Italy; 3 CNR Institute of Bioimages and Molecular Physiology, Section of Genoa, C/o Nuclear Medicine, IRCCS AOU San Martino-IST, 16132 Genoa, Italy; 4 Positron Emission Tomography Centre IRMET, Affidea, Turin, Italy; 5 SPIN Institute, CNR, Genoa, Italy; 6 ALS Center, “Rita Levi Montalcini” Department of Neuroscience, University of Turin, Turin, Italy; 7 AUO Città della Salute e della Scienza, Turin, Italy; 8 Department of Neuroscience, IRCCS San Martino IST, Genoa, Italy; 9 DINOGMI University of Genoa, Genoa, Italy; 10 Department of Mathematics (DIMA), University of Genoa, Genoa, Italy; 11 Medical Physics unit, IRCCS AOU San Martino-IST, Genoa, Italy; 12 Statistics and Epidemiology Unit, IRCCS AOU San Martino-IST, Genoa, Italy

**Keywords:** PET/CT, Amyotrophic lateral sclerosis, Spinal cord, Neuroimaging

## Abstract

**Purpose:**

In amyotrophic lateral sclerosis, functional alterations within the brain have been intensively assessed, while progression of lower motor neuron damage has scarcely been defined. The aim of the present study was to develop a computational method to systematically evaluate spinal cord metabolism as a tool to monitor disease mechanisms.

**Methods:**

A new computational three-dimensional method to extract the spinal cord from ^18^F-FDG PET/CT images was evaluated in 30 patients with spinal onset amyotrophic lateral sclerosis and 30 controls. The algorithm identified the skeleton on the CT images by using an extension of the Hough transform and then extracted the spinal canal and the spinal cord. In these regions, ^18^F-FDG standardized uptake values were measured to estimate the metabolic activity of the spinal canal and cord. Measurements were performed in the cervical and dorsal spine and normalized to the corresponding value in the liver.

**Results:**

Uptake of ^18^F-FDG in the spinal cord was significantly higher in patients than in controls (*p* < 0.05). By contrast, no significant differences were observed in spinal cord and spinal canal volumes between the two groups. ^18^F-FDG uptake was completely independent of age, gender, degree of functional impairment, disease duration and riluzole treatment. Kaplan-Meier analysis showed a higher mortality rate in patients with standardized uptake values above the fifth decile at the 3-year follow-up evaluation (log-rank test, *p* < 0.01). The independence of this value was confirmed by multivariate Cox analysis.

**Conclusion:**

Our computational three-dimensional method enabled the evaluation of spinal cord metabolism and volume and might represent a potential new window onto the pathophysiology of amyotrophic lateral sclerosis.

## Introduction

Neuroimaging with radionuclide methods allows the evaluation of many features of the central nervous system. However, while there is a large literature on the feasibility of nuclear medicine in the study of the brain, its use in the evaluation of the spinal cord (SC) has scarcely been investigated. This can at least partially be explained by the morphology of this nervous structure whose limited extension has prevented the development of standardized methods to systematically analyse its radioactivity uptake. To overcome this limitation – and to expand functional evaluation to the SC – we have recently developed a computational algorithm able to automatically recognize the spinal canal and SC in coregistered PET/CT images.

In the present study, we tested the accuracy and feasibility of this approach. As a first validation step, the method was applied to a series of patients with amyotrophic lateral sclerosis (ALS). Although this severe neurodegenerative disease actually causes progressive damage to the upper and lower motor neurons [1, 2], its evaluation by PET/CT imaging has been limited to the motor cortex. Several studies have shown the relevance of inflammatory signals in the motor cortex related to microglial activation or lymphocyte/macrophage infiltration [3–9] as possible correlates of disease phenotype [10]. However, virtually none of these studies systematically evaluated SC damage.

Extending the analysis to the lower motor neurons might thus be a new approach to improving our understanding of the heterogeneous nature of ALS. The clinical course of this severe disease is variable with survival usually averaging 3 – 4 years from symptom onset [11] or more than 10 years in a small number of patients [12]. As a consequence, our computational algorithm and its potential to objectively define the severity of SC involvement might complement the clinical evaluation in defining disease aggressiveness.

## Materials and methods

The study included 30 patients (20 men, mean age 66 ± 10 years, median 69 years, range 34 – 82 years) with definite, probable or probable laboratory-supported spinal onset ALS according to the revised El Escorial criteria [13]. All subjects provided signed informed consent to be entered into the study that was approved by the Ethics Committees of IRCCS AOU San Martino-IST in Genova and of AUO Città della Salute e della Scienza in Torino, Italy. The revised ALS functional rating scale (ALSFRS-R, maximum score 48) [14] was used to evaluate overall patient functional status at the time of the study.

Data obtained in the patients were compared with data from 30 control subjects without any history of neurodegenerative disease, randomly selected from a previously published normalcy database [15] according to a case-control criterion considering, age, sex and scanner used. This registry includes patients who had a negative PET/CT scan 2 years after surgery for a nonulcerated lesion, with a Breslow thickness less than 1 mm and negative sentinel node biopsy. Overall 10-year survival approaches 100 % in this population [16]. However, to minimize the possible inclusion of cancer patients, enrolment was delayed by 2 years after scanning so as to include only those subjects who did not have any evidence of relapse or any clinical event during this follow-up period.

### PET/CT imaging

All subjects were studied in the early morning after fasting for 12 h. Serum glucose was assessed to ensure a glucose level ≤2 g/l. A bolus injection of FDG was administered (4.8 – 5.2 MBq/kg body weight) with the patient lying in the supine position in a quiet room and instructed not to move or talk. A three-dimensional (3D) whole-body scan (arms down position) was started 60 – 75 min after tracer administration using an integrated PET/CT scanner (Hirez, Siemens Medical Solutions; or Discovery, GE Healthcare).

Raw PET data were reconstructed into a 128 × 128 matrix using a 3D iterative reconstruction algorithm (ordered-subsets expectation maximization, three iterative steps, eight subsets). Postfiltering with a 3D gaussian filter and scatter correction were applied, and attenuation correction was performed using the CT data. The resulting resolution was 4.0 mm full-width at half-maximum for both scanners. The two imaging systems were cross-calibrated according to the standard procedures for quality control. In both centres, the PET scanners were periodically calibrated with the same phantom, i.e. a cylinder of 20 cm outer diameter and 20 cm length filled with a solution containing 100 MBq of ^18^F-FDG that was counted for 1 h to minimize the statistical noise, while images were reconstructed with the same algorithm used for the clinical protocol.

The entire CT dataset was coregistered with the 3D PET images using commercially available software interfaces. For each patient, ideal body weight was calculated according to the conventional formula of Robinson et al. [17]. The spinal canal and SC were segmented from the low-dose CT data acquired during the PET/CT scan to confirm the performance of the analysis in a broad clinical setting.

### Image analysis

The different spinal canal and SC districts were defined anatomically considering the cervical segment as the region between the skull base and the plane adjacent to the caudal face of the C7 vertebral body. The dorsal segment was defined as the district between this plane and the one adjacent to the caudal face of D12. The sacral and lumbar canal districts were a priori considered free from SC and were thus excluded from the analysis.

Images were analysed according to a previously validated method [18, 19] based on generalization of the Hough transform technique for pattern recognition [20]. According to the original definition [21], given a point $P=\left({x}_P,{y}_P\right)$ in the image plane satisfying the equation of a straight line

1 $$ y= ax+b $$

the Hough transform of *P,* with respect to the class of straight lines, is the straight line of equation

2 $$ {y}_P=a{x}_P+b $$

into the parameter space where the two independent real parameters *a*, *b* vary [21]. This definition implies that all points on the line (1) in the image space correspond to lines in the parameter space that all intersect at the point (*a, b*) uniquely identifying the original line. This correspondence between the image and the parameter spaces holds not only for lines, but also for several classes of algebraic curves. This simple fact inspired the following pattern recognition algorithm for the identification of curves in a digital image:

1. Apply the traditional Canny edge detection algorithm [22] to extract discontinuities.
2. Compute the Hough transforms with respect to the selected family of curves of all points in the image plane highlighted by the edge detection process.
3. Discretize the parameter space into cells whose size is optimized for the specific recognition problem (for the recognition of SC the four-dimensional parameter space is discretized with hypercubes with side equal to 0.02, while for the recognition of the spinal canal the two-dimensional parameter space is discretized with squares with side again 0.02).
4. Construct an accumulator function defined on the discretized parameter space such that for each cell the value of the accumulator function is equal to the number of Hough transforms passing through that cell.
5. Search for the parameter values identifying the cell where the accumulator function reaches its maximum.

**Fig. 1:**
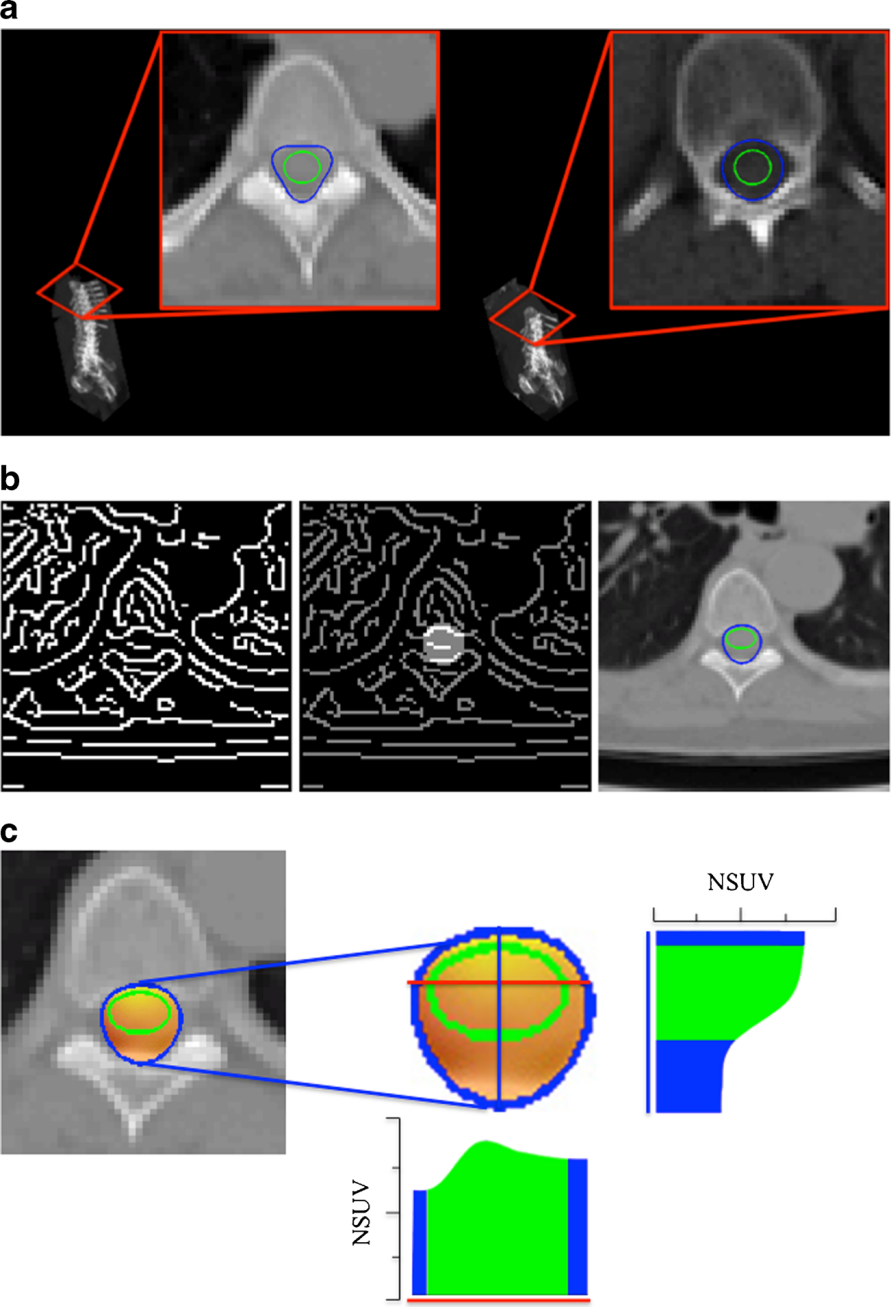
**a** Detection of spinal canal and spinal cord at different heights of the vertebral column. The Hough transform-based procedure with respect to the curve with three convexities allows identification of the spinal canal (*blue line*), while the spinal cord is detected by the Hough transform-based procedure with respect to the ellipse (*green line*). **b** *Left to right*: edge detection of a CT slice in the vertebral region; edge points inside the region bounded by the curve with three convexities; and the curve with three convexities (*blue line*) and the ellipse (*green line*) detected by applying the Hough transform-based procedure. **c** *Left to right*: fusion of CT and PET data for the slice considered in **b** zoomed on the spinal region, and histograms of the behaviour of the normalized standardized uptake values for the same slice and along two specific projections highlighted in *red*

We applied this scheme to the recognition of both the spinal canal and the SC districts in whole-body CT images of control subjects and ALS patients. Specifically, the family of curves with three convexities, represented by the equation

3 $$ {C}_{a,b}:\ {\left({x}^2+{y}^2\right)}^3={\left(a\left({x}^2+{y}^2\right)-b\left({x}^3-3x{y}^2\right)\right)}^2, $$

was particularly appropriate for the optimal detection of the spinal canal (Fig. 1).

By contrast, the four-parameter family of ellipses expressed in the form

4 $$ {E}_{a,b,c,d}:\ {b}^2{\left(x-c\right)}^2 + {a}^2{\left(y-d\right)}^2-{a}^2{b}^2=0 $$

was the best candidate for identifying the SC district. In agreement with our previous experience [18, 19], visual inspection by an experienced nuclear medicine physician always confirmed the reliability of the segmentation algorithm.

For each CT slice, the two curves identifying the spinal canal and SC were used to create two sets of binary masks with one zero outside and one inside each curve, respectively. These masks were multiplied against the coregistered PET slice in order to digitally extract the metabolic information represented as standardized uptake value (SUV) of local FDG radioactivity [23]. Finally, the average SUVs of both spinal canal and SC were divided by the corresponding average SUV in the liver in order to account for possible differences in scanner sensitivity to obtain the normalized SUVs (NSUVs). NSUVs of the whole SC were computed according to the formula:

5 $$ SC\_ NSUV = \frac{\left(C\_ NSUV \times cervical\ SC\  volume\right)+\left(D\_ NSUV \times dorsal\ SC\  volume\right)}{cervical\ SC\  volume+ dorsal\ SC\  volume}, $$

where C_NSUV and D_NSUV indicate the average NSUV of the cervical and dorsal SC segments, respectively.

### Statistical analysis

All data are reported as means ± SD. Unpaired or paired *t* tests were used, as appropriate. Linear regression analysis was performed using the least squares method. A *p* value <0.05 was considered significant.

The 30 ALS patients were divided into two groups using the fifth decile NSUV (0.67). Survival of these two groups was analysed using the Kaplan-Meier method and compared using the log-rank test. To assess the prognostic relevance of the NSUV of the SC (SC_NSUV), a set of univariate and multivariate Cox proportional hazard models were fitted to the data. In the univariate analysis, the incidence of death was modelled as a function of each of the following variables: age, sex, time from ALS diagnosis to PET/CT scan, riluzole therapy, ALS functional score and average SC_NSUV (below and above the fifth decile). Then all six variables were tentatively included in a multivariate Cox model by means of a step-down (backward) procedure, based on the likelihood ratio test: variables with a *p* value >0.1 were removed from the model. Proportionality assumptions were assessed as previously described [24].

## Results

### Clinical characteristics of the patients and controls

The main clinical findings in the ALS patients and control subjects are shown in Table 1. According to the case-control selection criterion, age, sex, and ideal body weight were similar in the two groups. Similarly, serum glucose levels were comparably low in the two groups (Table 1). The ALSFRS-R score, updated for each patient on the day of imaging, ranged from 20 to 46/48.

**Table 1:** Demographic characteristics of patient populations

Characteristic	Control subjects	ALS patients
Age (years)		
Mean ± SD	60 ± 13	66 ± 11
Median (range)	59 (33 – 79)	69 (34 – 82)
Number of subjects		
Men	20	20
Women	10	10
Ideal body weight (kg), mean ± SD	66 ± 9	66 ± 9
Time from ALS diagnosis to PET/CT (months)		
Mean ± SD	–	18 ± 15
Median (range)	–	17 (2 – 69)
ALS functional rating scale score		
Mean ± SD	–	39 ± 5
Median (range)	–	39 (20 – 46)
Riluzole therapy, *n*	–	21
Serum glucose (mg/dL)		
Mean ± SD	98 ± 13	102 ± 12
Median (range)	100 (73 – 130)	98 (69 – 129)
Follow-up after PET/CT (months)		
Mean ± SD	24	14 ± 7
Median (range)	–	16 (1 – 36)
Number of deaths during follow-up	0	13

The time from ALS onset to PET/CT imaging was 18 ± 15 months (range 2 – 69 months, median 16 months). The patients were followed up for 1 – 36 months after imaging (median 14 months). During this period, 13 patients died from respiratory complications (Table 1).

### Shape and volume of spinal canal and spinal cord

The spinal canal profile significantly changed across the whole stack of CT images. However, the Hough transform method allowed the parameters of the curve with three convexities to automatically change allowing the curve to optimally adapt itself to the profile. The profile of the SC could be recognized by the Hough transform of an ellipse for all spinal districts. Further, the parameters of such an ellipse changed less than 10 % in the cervical district and less than 12 % in the dorsal district. This formulation did not change throughout the spinal districts and the SC was segmented by adjusting the numerical parameters.

**Fig. 2:**
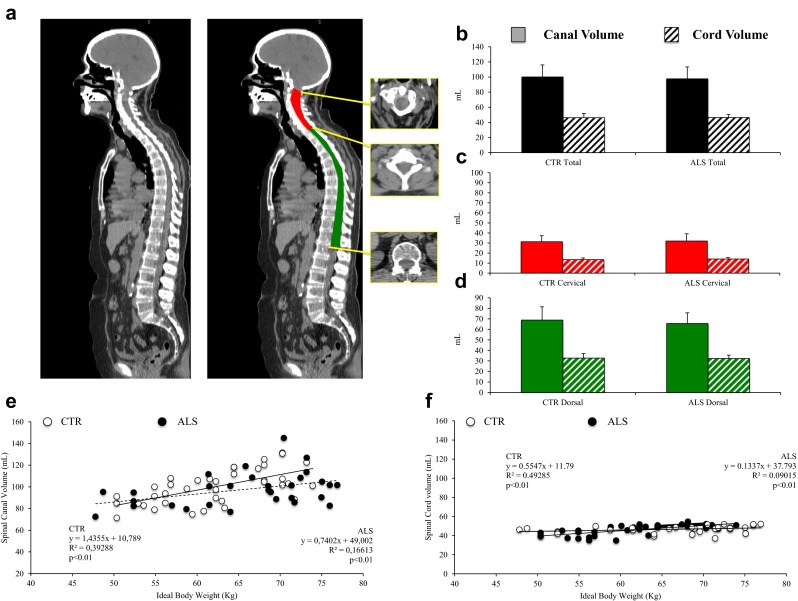
**a** Sagittal whole-body CT scan (*left*) and the corresponding image showing extraction of the cervical (*red*) and dorsal (*green*) spinal canal. **b–d** Volumes of the spinal canal (*solid bars*) and spinal cord (*hatched bars*) in the control subjects (*CTR*) and in the 30 ALS patients (*ALS*): **b** total volumes, **c** cervical region, **d** dorsal region. There are no differences between the control subjects and ALS patients. **e**, **f** Linear regression analysis of the relationship between spinal canal volume (**e**) and spinal cord volume (**f**) and ideal body weight

As shown in Fig. 2, the disease did not affect the anatomical features of the spinal canal or the SC. The spinal cord volume was similar in ALS patients and in control subjects, both in the cervical district (32.08 ± 7.12 mL vs. 31.36 ± 5.99 mL, respectively, *p* = 0.79) and in the dorsal district (65.60 ± 10.09 mL vs. 68.84 ± 12.61 mL, respectively, *p* = 0.10,; Fig. 2a–e).The extracted SC volume was also similar in patients and control subjects both in the cervical segment (13.99 ± 1.42 mL vs. 13.53 ± 1.60 mL, respectively, *p* = 0.47, ns) and in the dorsal segment (32.60 ± 3.22 mL vs. 32.81 ± 4.25 mL, respectively, *p* = 0.56; Fig. 2b). As for the spinal canal, ideal body weight was directly correlated with overall SC volume in control subjects, but the significance of this relationship was lower in ALS patients (Fig. 2f).

### Spinal canal and spinal cord metabolic activity

**Fig. 3:**
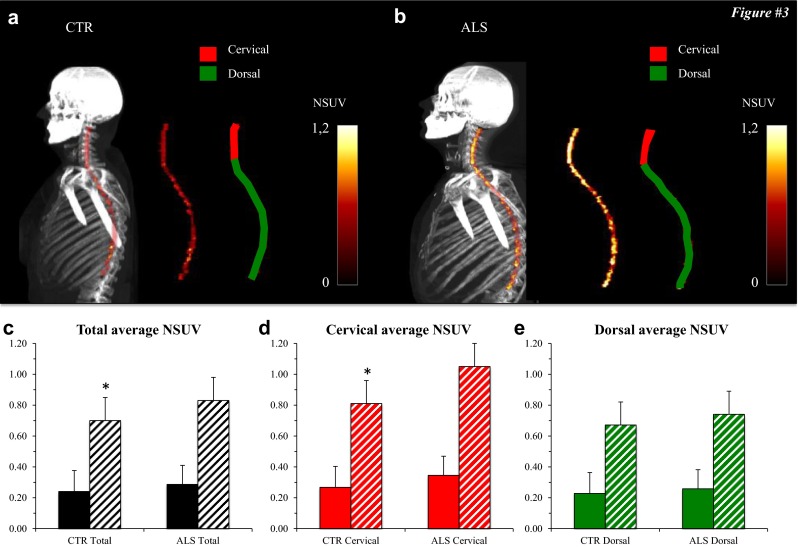
**a**, **b** Whole-body maximum intensity projection CT images coregistered with the extracted PET data for the spinal cord in a control subject (**a** *CTR*) and an ALS patient (**b** *ALS*). The PET data alone are also shown for the corresponding SCs as the average SUV normalized to the corresponding liver values (average NSUV) together with images of the cervical and dorsal segments. **c–e** Average NSUV for the spinal canal (*solid bars*) and spinal cord (*hatched bars*) in control subjects (*CTR*) and 30 ALS patients (*ALS*): **c** whole spinal cord/spinal canal, **d** cervical segment, **e** dorsal segment. FDG uptake in the spinal cord was significantly lower in control subjects for the whole spinal cord (**c**) and for the cervical segment (**d**), but not for the dorsal segment (**e**). *p < 0.05

**Fig. 4:**
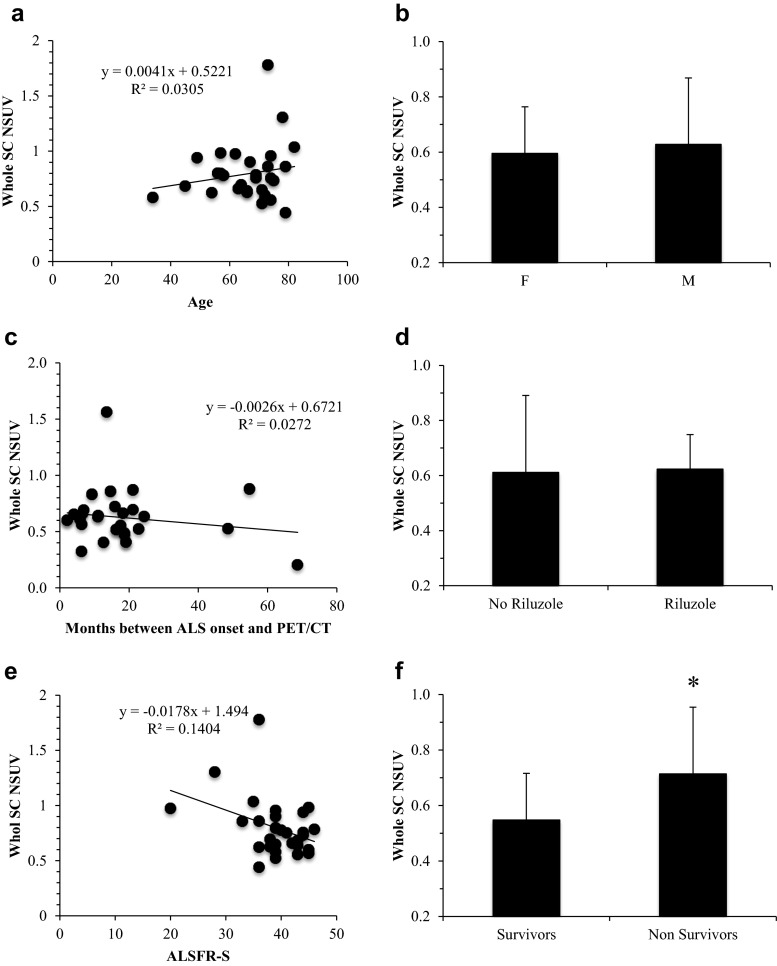
**a**, **c**, **e** Linear regression analysis of the relationship between spinal cord (*SC*) NSUV and patient age (**a**), time between diagnosis and imaging (**c**) and revised ALS functional rating scale (*ALSFR-S*) score (**e**). **b**, **d**, **f** The spinal cord metabolic pattern (in terms of NSUV) was not significantly different between male (*M*) and female (*F*) patients (**b**), or between those treated and those not treated with riluzole (**d**), but SC NSUV was significantly higher in patients who had died (*Nonsurvivors*) than in those who were still alive (*Survivors*) at the end of the 36-month follow-up period (**f**). *p < 0.05

On visual inspection, the radioactivity distribution within the SC was relatively homogeneous without any focal areas of enhanced uptake in both control subjects and ALS patients (Fig. 3a, b). As shown in Fig. 3c–e, the average NSUV was not significantly different between the two groups in the whole spinal canal or in the cervical and dorsal districts. As expected, the average NSUV was significantly lower than the corresponding SC_NSUV in the same districts in both groups (Fig. 3c). By contrast, the radioactivity distribution in the SC showed a different pattern. FDG uptake in the SC was higher in ALS patients than in controls. This difference in FDG uptake between the groups was statistically significant for the whole SC (NSUV 0.82 ± 0.28 in ALS patients vs. 0.70 ± 0.14 in controls, *p* < 0.05) and for the cervical segment (NSUV 0.99 ± 0.37 vs. 0.85 ± 0.20, respectively; *p* < 0.05, Fig. 3c, d), but not for the dorsal segment (NSUV 0.72 ± 0.24 vs. 0.62 ± 0.18, respectively; *p* = 0.08; Fig. 3e).Interestingly, the effect of ALS on FDG accumulation in the SC was independent of demographic and clinical variables. In fact, SC_NSUV did not correlate with age, sex, ALSFRS-R score, time elapsed from diagnosis to PET/CT or riluzole treatment (Fig. 4).

### Spinal cord metabolic pattern and patient outcomes

**Fig. 5:**
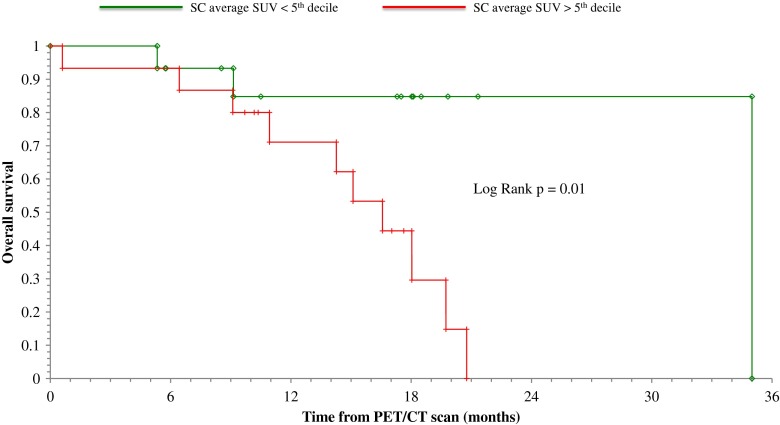
Kaplan-Meier curves showing overall survival in patients with SC_NSUV above and below the fifth decile. High FDG uptake in the whole spinal cord was associated with a higher mortality rate

The potential prognostic value of metabolic information was suggested by the observation that the 13 patients who died showed a higher SC_NSUV than the 17 patients who were still alive at 36 months (0.71 ± 0.26 vs. 0.55 ± 0.16, respectively, *p* < 0.05; Fig. 4f). This result was confirmed by Kaplan-Meier analysis showing that the 16 patients with NSUV above the fifth decile had a significantly higher mortality rate than those with NSUV below the fifth decile at 36 months after PET/CT imaging (log-rank test, *p* < 0.01; Fig. 5).

In the univariate analysis, the only predictive variable for overall survival was SC_NSUV (Table 2). The multivariate analysis confirmed the independent prognostic value of the SC metabolic pattern (hazard ratio, HR, 24.3, 95 % confidence interval, CI, 2.2 – 262.8) although the strength of the association needs to be confirmed in a large sample. Prognosis was not associated with age, ALSFRS-R score, time from diagnosis to PET scanning or the presence/absence of riluzole treatment. A significantly increased risk of death was found for male gender (HR 4.43, 95 % CI 0.87 – 22.5), and with borderline significance with age (*p* = 0.055) and ALSFRS-R score (*p* = 0.082), but there was no trend in risk with increasing or decreasing age or ALSFRS-R score. On the other hand, there was no significant difference in ALSFRS-R score at the time of PET scanning between those who had died and those who were still alive at 36 months (39.61 ± 3.5 vs. 38.76 ± 6.66, *p* = 0.7).

**Table 2:** Prediction of death

Variable	Outcome results^a^			Univariate analysis				Multivariate analysis			
	No. of patients	No. of deaths	Mortality rate (%)	Hazard ratio	95 % confidence interval	*p* value		Hazard ratio	95 % confidence interval	*p* value	
						For heterogeneity	For trend			For heterogeneity	For trend
Age (three categories)											
≤63 years	10	3	30	1 (reference)	–	0.213	0.810	1 (reference)	–	0.055	0.226
64 – 72 years	10	4	40	3.20	0.74 – 13.84			7.45	1.20 – 46.37		
≥73 years	10	6	60	1.22	0.24 – 6.09			6.56	0.45 – 95.11		
ALS score (three categories)											
≤38	10	4	40	1 (reference)	–	0.323	0.280	1 (reference)	–	0.082	0.374
39 – 42	10	4	40	1.39	0.35 – 5.51			2.06	0.36 – 11.64		
≥43	10	5	50	0.43	0.09 – 1.98			0.30	0.04 – 2.15		
Time from diagnosis to PET/CT (three categories)											
≤9 months	9	6	67	1 (reference)	–	0.367	0.240	–^b^	–	0.86	–
10 – 19 months	14	3	21	0.41	0.10 – 1.74			–	–		
≥20 months	7	4	57	0.45	0.11 – 1.86			–	–		
Riluzole treatment											
No	9	4	44	1 (reference)	–	0.488	–	–^b^	–	0.51	–
Yes	21	9	43	1.54	0.45 – 5.27			–	–		
Sex											
Female	10	4	40	1 (reference)	–	0.272	–	1 (reference)	–	0.049	–
Male	20	9	45	2.02	0.54 – 7.56			4.43	0.87 – 22.55		
Average normalized spinal cord SUV (>5th decile)											
≤0.67	16	3	19	1 (reference)	–	0.003	–	1 (reference)	–	0.01	–
>0.67	14	10	71	6.87	1.50 – 31.58			24.3	2.25 – 262.77		

^a^Total 30 patients, 13 died, overall mortality rate 43 %

^b^Removed from the final Cox multivariate model (*p* > 0.1)

## Discussion

The present study is the first attempt to estimate the metabolic pattern of the spinal canal and SC from whole-body PET/CT images. The results obtained indicate that this computational approach might be a new method for exploring the status of the SC in different conditions, besides its potential to complement the routine analysis of ALS patients.

### Recognition and measurement of spinal canal and spinal cord by CT analysis

Estimation of the canal space throughout the whole spine was based on the assumption that compact bone is the human tissue with the highest X-ray attenuation coefficient and can thus be easily identified and extracted in each CT slice. This computational approach is commonly used in commercially available software for 3D representation of the skeleton, and its potential has been previously validated in our laboratory for the characterization of intraosseous volume and bone marrow metabolic activity [15, 25, 26]. However, thresholding the Hounsfield values on the CT images is not effective in this application since the spinal canal is not directly surrounded by osseous tissue in all slices. To overcome this problem, we developed a pattern recognition method based on the Hough transform that permitted determination of canal shape also in segments between the different vertebral bodies. This approach permitted extraction of PET data and analysis of FDG uptake throughout the whole SC in a systematic fashion.

The most relevant feature of our algorithm is its fully deterministic nature with the user being asked to identify only the occipital skull border and the caudal face of D12. This plane was arbitrarily set as the caudal edge of the SC because of the limited resolution of CT images that prevented accurate evaluation of more distal segments. On the other hand, the operator-independent nature of this method virtually abolished the need for statistical analysis of its reproducibility measured in terms of either interobserver or intraobserver variability.

### FDG and spinal cord involvement in ALS

As a first validation step, we applied this method to a cohort of ALS patients. This model fitted our purpose due to the pathological evidence of significant damage to the SC neurons [1, 2]. By contrast, spinal onset ALS was associated with a slight yet significant increase in SC uptake of FDG. Several considerations support the concept that this finding reflects an increased metabolism of SC structures. First, the increase in FDG uptake was most evident in the cervical spine segments. Secondly, it was not related to abnormal regulation of serum glucose levels [27]. Finally, the difference between controls and ALS patients virtually disappeared when the whole spinal canal was considered, most likely because of both the smoothing effect of the large volume and the spillover of radioactivity uptake into surrounding outside tissues.

The increased FDG uptake in ALS SC at least partially conflicts with the expected reduction in tissue metabolic rate caused by the neuronal loss that has been described not only in the motor cortex but also in the anterior horns of the SC in ALS patients. Similarly, it partially disagrees with the emerging prognostic value of frontal hypometabolism in subgroups of ALS patients [4, 5, 28]. Nevertheless, it has been extensively documented in the literature that neuroinflammation is a key-signalling event in ALS [10]. This concept originated from pathology studies showing activation of microglia and astrocytes, as well as the presence of lymphocytes and macrophages in post-mortem tissue from the motor cortex and SC of both patients and experimental models of ALS [29, 30]. As a common interpretation, these studies suggested that activated microglia might accumulate within the degenerating areas and might contribute to propagating and sustaining the tissue damage through the release of free radicals and other neurotoxic substances such as glutamate [30–32]. More recently, this mechanism has been shown to also occur in the early disease phases. In fact, different studies have shown inflammatory microglial activation in various cortical areas of ALS patients using PET imaging and different tracers targeting the translocator protein TSPO [6–8]. In this line, our observation of a relative increase in SC FDG uptake might extend the previously documented pattern of motor cortex damage in ALS to lower motor neurons and might reflect inflammatory mechanisms rather than the expected consequences of motor neuron loss and subsequent SC atrophy.

FDG uptake cannot be considered per se a specific marker of microglia activation. Nevertheless, the relevance of inflammatory mechanisms on ALS progression is supported by the follow-up evaluation. Indeed, the Kaplan-Meier analysis indicated that higher FDG uptake significantly predicted a higher mortality rate. The multivariate analysis confirmed this finding and showed the independent prognostic value of SC metabolism. The observed difference in prognosis between patients with high and low FDG uptake was striking (HR 24). Nevertheless, the effective clinical potential of SC FDG uptake in outcome prediction could not be assessed in the present study because of the limited number of patients, the retrospective nature of the evaluation and particularly the fact that this hypothesis was generated by our study and needs to be validated on independent datasets.

 The fact that there were 17 patients censored before death does not imply any risk of bias because these were not patients lost to follow-up, but patients with recent enrolment. Furthermore, they were similarly distributed in the two groups of patients with SUV below and above the median. A similar consideration also applies to the exclusion of patients with bulbar onset disease: this decision – justified by the focus of our computational algorithm on SC metabolism – prevents the ability to define the clinical value of this information. Finally, the relatively small number of patients studied together with the exclusion of subjects with bulbar onset ALS prevented verification of the potential added value of SC metabolism with respect to brain FDG uptake. Nevertheless, if confirmed in larger prospective studies, the prognostic significance of SC metabolic pattern would indicate a relevant role for SC inflammatory response in ALS progression.

In conclusion, the present study showed the potential of Hough transform in delineating the spinal canal and SC in clinical PET/CT scanning. As a first validation step, this method was applied to a cohort of ALS patients as a model of pathologically confirmed damage to the SC neurons. However, its use can obviously be extended to different conditions in which the possibility of extracting the spinal canal and its contents might be a useful tool to precisely evaluate the site of SC injury from whatever cause and particularly to improve the accuracy in monitoring its evolution. In this setting, the proposed computational approach to PET/CT images would permit the limitations of visual inspection to be overcome, limitations that have so far hampered the evaluation of SC damage in patients with inflammatory diseases [33], posttraumatic conditions [34], cancer infiltration [35] or autoimmune/autotoxicity disorders [36]. Similarly, its use in combination with tracers selectively targeting specific neuronal functions might contribute to the understanding of SC involvement in different diseases.

As far as ALS is concerned, the availability of this biomarker and its operator-independent nature could be invaluable for the development of new therapeutic approaches, especially in early phase clinical trials in which current entry criteria that consider only phenotype, disability severity and disease duration markedly hamper the correct identification of target patients.
